# Children’s outdoor active mobility behaviour and neighbourhood safety: a systematic review in measurement methods and future research directions

**DOI:** 10.1186/s12942-020-00254-w

**Published:** 2021-01-07

**Authors:** Roula Zougheibe, Jianhong (Cecilia) Xia, Ashraf Dewan, Ori Gudes, Richard Norman

**Affiliations:** 1 School of Earth and Planetary Sciences, Curtin University, Kent Street, Perth, WA 6102 Australia; 2 School of Public Health and Community Medicine, UNSW Medicine, Sydney, NSW, Australia; 3 School of Public Health, Curtin University, Perth, WA Australia

**Keywords:** Children’s active mobility, Perceived safety, Measured crime, Geographic information system (GIS), Global positioning system (GPS), Activity tracking, Spatiotemporal analysis, Methodological conceptual framework

## Abstract

**Background:**

Numerous studies have examined the association between safety and primary school-aged children’s forms of active mobility. However, variations in studies’ measurement methods and the elements addressed have contributed to inconsistencies in research outcomes, which may be forming a barrier to advancing researchers’ knowledge about this field. To assess where current research stands, we have synthesised the methodological measures in studies that examined the effects of neighbourhood safety exposure (perceived and measured) on children’s outdoor active mobility behaviour and used this analysis to propose future research directions.

**Method:**

A systematic search of the literature in six electronic databases was conducted using pre-defined eligibility criteria and was concluded in July 2020. Two reviewers screened the literature abstracts to determine the studies’ inclusion, and two reviewers independently conducted a methodological quality assessment to rate the included studies.

**Results:**

Twenty-five peer-reviewed studies met the inclusion criteria. Active mobility behaviour and health characteristics were measured objectively in 12 out of the 25 studies and were reported in another 13 studies. Twenty-one studies overlooked spatiotemporal dimensions in their analyses and outputs. Delineations of children’s neighbourhoods varied within 10 studies’ objective measures, and the 15 studies that opted for subjective measures. Safety perceptions obtained in 22 studies were mostly static and primarily collected via parents, and dissimilarities in actual safety measurement methods were present in 6 studies. The identified schematic constraints in studies’ measurement methods assisted in outlining a three-dimensional relationship between ‘what’ (determinants), ‘where’ (spatial) and ‘when’ (time) within a methodological conceptual framework.

**Conclusions:**

The absence of standardised measurement methods among relevant studies may have led to the current diversity in findings regarding active mobility, spatial (locality) and temporal (time) characteristics, the neighbourhood, and the representation of safety. Ignorance of the existing gaps and heterogeneity in measures may impact the reliability of evidence and poses a limitation when synthesising findings, which could result in serious biases for policymakers. Given the increasing interest in children’s health studies, we suggested alternatives in the design and method of measures that may guide future evidence-based research for policymakers who aim to improve children’s active mobility and safety.

## Introduction

Extensive evidence supports that high levels of physical activity (PA) have profound long-term health benefits for children [1–5]. Among primary school-aged children, outdoor active (non-motorised) forms of mobility such as free play, organised sport or active travel between destinations are significant contributors to children’s overall PA [6]. Within the field of child-specific research, one well-established potential influence on children’s active behaviour is neighbourhood safety in terms of personal safety and road dangers [7, 8]. Although, the effects of neighbourhood safety on PA may vary according to whether dangers are measured or perceived [7], neighbourhood safety continues to threaten children’s PA [9] and affect their activity space [10]. However, existing literature has produced mixed findings on the effect of safety on children’s PA [11, 12]. Inconsistency in methods of measures may largely explain the disagreements between findings. For example, perceived safety has often been assessed via different questionnaires developed to fit the objectives of individual studies [13]. Moreover, measuring children’s forms of active mobility behaviour have varied across studies from parent or child questionnaires [14] to travel diaries [15] to time-latitude–longitude records of child mobility [16]. Additionally, a child’s neighbourhood—a place that offers opportunities for the majority of children's daily routine activities (i.e., where they live, go to school, visit a destination or play)—is seen as playing a crucial role in the outcomes of the examined contextual determinants of child health behaviour [10].

The emerging tools that measure the intensity of movement (e.g., accelerometers) or the geographic (spatial) location of movement (e.g., global positioning systems [GPS]), and geographic technology, have proven advantageous in improving our understanding of evidence-based child-related research [17]. However, the pathway to obtaining reliable data for analysis remains laden with challenges in terms of measurement, integration and technological limitations.

The methodological reviews of safety exposures in child-related studies are scarce. Available manuscripts have either addressed broad age groups combining environmental determinants measurements methods or were specific [18–21]. A rigorous synthesis of methodological measures based on an interdisciplinary vision in research examining neighbourhood safety effects on active mobility behaviour is still lacking. More importantly, variations in active behaviour from childhood to adolescence [22] suggest that measurement methods may vary by age group. Thus, the dearth of review in measures of active behaviour in safety context and the profound importance of this age group [31] on lifelong PA have directed our focus onto primary school-aged children. We expand upon the earlier approach and examine children outdoor active mobility behaviour (COAMB) in terms of free play, scheduled activities and the associated active travel between destinations, as opposed to more typical reviews that only discuss one type of PA, such as trips to and from school or free play [23].

In this review, we aim to synthesis and assess the different methodological measures found in studies that examined the impact of safety exposures on modifying primary school-aged COAMB. To accomplish this goal, this systematic review seeks to answer three questions: First, where does current research stand in terms of measurement methods used to examine the effect of neighbourhood safety perceived (by parents or children) and measured (actual), on primary school-aged children? Secondly, what are the gaps in current practice? Finally, what are the future directions that could be taken in the area to better inform the decision-making process?

## Methods

A systematic literature search was conducted based on the methodological guidelines of the Preferred Reporting Items for Systematic Reviews and Meta-Analyses (PRISMA) statement checklist of 27 items [24]. However, some items on the list were unchecked as they do not apply to a methodological review (Additional file 1). No protocol was published for this literature review.

### Eligibility criteria

To be included in the review, papers had to meet the following criteria: be peer-reviewed, be published in English, report participants’ characteristics (size, age, etc.) examined primary school-aged children, measure forms of children’s outdoor active mobility (i.e., PA, outdoor active playing, walking, running, biking and active travel), and address (a) perceived or (b) measured (actual) correlations with safety (personal and road). Reports, theses, protocols, non-peer-reviewed studies and studies that assessed the effect of interventions (e.g., traffic calming) were excluded. Detailed inclusion and exclusion criteria used for this review are also listed in Additional file 2.

### Search strategy

The literature search began in February 2019 based on the above eligibility criteria. Six electronic databases, Google Scholar, PubMed, Scopus, ScienceDirect, SpringerLink and Web of Science were searched. Works were retrieved using combinations of search terms explicitly developed to meet the objectives of this review. We used a combination of a minimum of three keywords rotated in turn, with each belonging to one of the following groups: (1) the target population, AND (2) active behaviour, AND (3) the safety neighbourhood’s exposures, OR (4) moderators and mediators OR (5) spatiotemporal aspects. Details of terms used per group are found in Additional file 2.

### Selection process

The primary author (RZ) carried out a comprehensive screening of the retrieved studies. The final date of the search was July 2020. Furthermore, RZ also scanned the reference lists of the individual papers to identify further studies. An independent screening of abstracts for inclusion was conducted by the second author (CX). The third author (RN) resolved disputes regarding which studies to include.

### Data extraction

After the study selection, RZ organised and extracted the relevant data into three main categories: (1) study characteristics, namely the author(s), year of publication, study location and participant demographics (e.g. age, gender and ethnicity); (2) measures of safety (perceived and/or actual) and children’s outdoor active mobility behaviour (COAMB); and (3) neighbourhood measures. We extracted the studies’ tools and methods for measuring safety, COAMB and neighbourhoods, as well as the significant results, study variables and methods used to examine the relationships between these factors.

### Methodological assessments of individual studies quality

A formal assessment of the included studies’ quality was completed independently and critically by two reviewers RZ and BJ. Any rating discrepancies were discussed, and a shared decision was reached in required cases. RZ compiled a 20 priori methodological quality criteria, of which 13 were adopted from earlier reviews [20, 25–27]. The remaining seven criteria expanded upon measurement methods that were believed to be fundamental when examining the effect of safety exposures on COAMB (see Additional file 2: Table 1). Each study was allocated a point if a criterion was present or was allocated no points if the criteria were absent or inadequately described; if a criteria were not applicable, it was discounted from the total score. Each study could score a total of 8 points, and this maximum score was used to calculate a percentage of study quality [25]. Study quality was rated robust if the study secured a percentage of ≥ 66.7%, was rated fair if it scored between ≥ 50 and < 66.6% or was rated weak if it scored < 50% [25]. Further details on quality assessment can be found in Additional file 2.

## Results

### Study selection

**Fig. 1:**
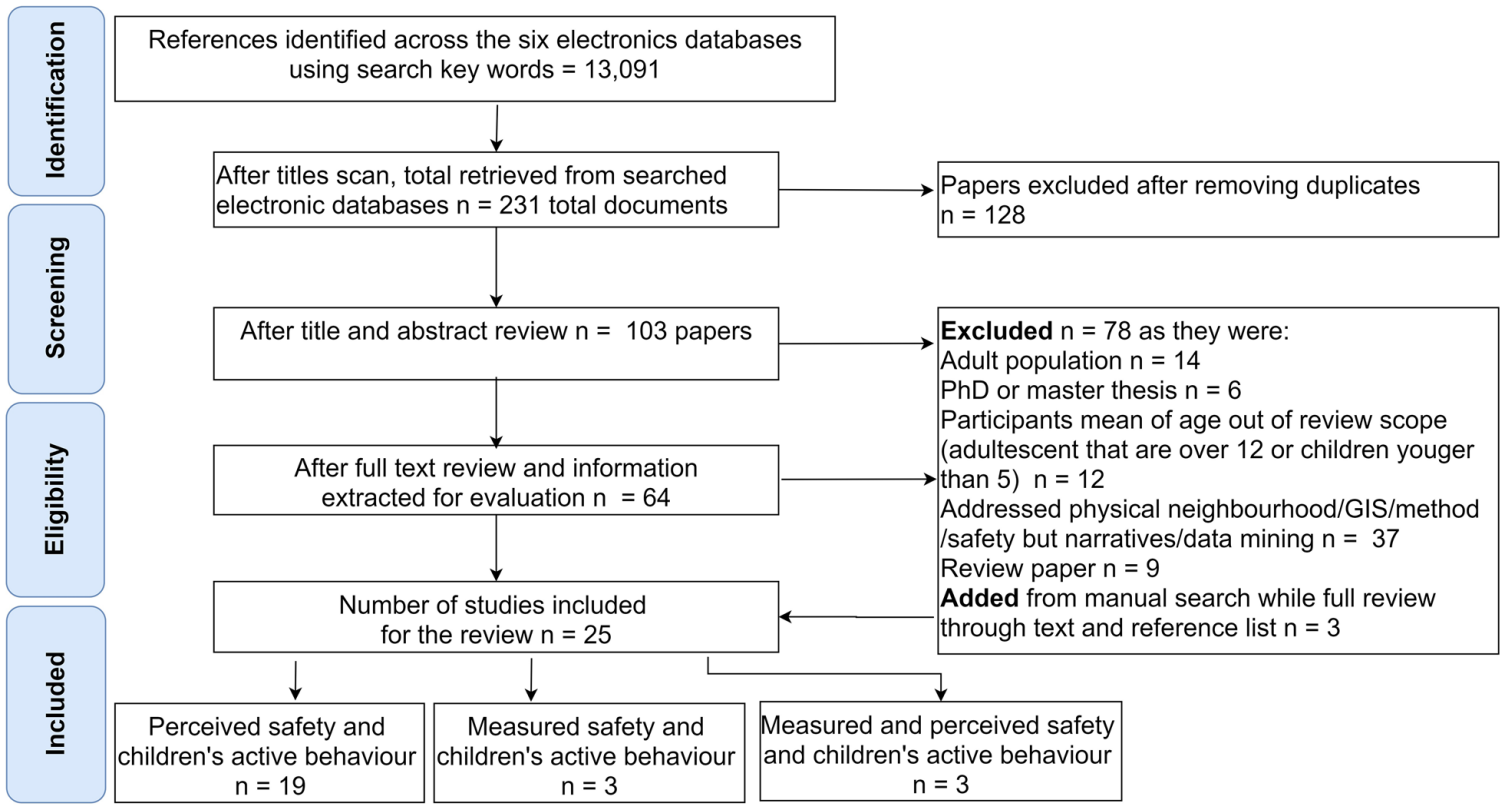
Flow chart of the PRISMA for the systematic methodological review

The title scan that was carried out using the defined key terms has identified 231 papers, out of 13,091 title references across six databases, as potentially relevant. After 128 duplicates were removed, the remaining 103 articles’ titles and abstracts were screened, and 64 studies were thoroughly reviewed. A manual search of the reference lists of the individual studies yielded three additional studies. A total of 25 articles passed a thorough review and met the inclusion criteria (see Fig. 1).

### Study characteristics

Aside from three longitudinal studies, 22/25 studies (88%) had a cross-sectional design. Sample sizes ranged from 35 [28] children to 3,200 children [29]. Forms of COAMB were examined in correlation to perceived safety (19/25 studies; 76%), measured safety (3/25 studies; 12%) or both measured and perceived safety (3/25 studies; 12%). Multiple studies (10/25 studies; 40%) extracted data for their analysis from larger projects (e.g. the Personal and Environmental Association with Child’s Health [PEACH; UK] project), see Table 1. With the exception of two Iranian manuscripts [30, 31], 23/25 studies (92%) explored populations in developed countries. These 23 studies were geographically distributed to include five studies each in the United States and the United Kingdom; four in Canada; three in Australia; two in New Zealand; and one each in Portugal, Finland, the Netherlands and Austria (Additional file 2: Figure 1).

**Table 1:** Summary of characteristics and methods of measures to examine the association between children outdoor active behaviour and neighbourhood safety in the included studies

#	Study characteristics		Studies’ measures					Analysis method
	Citation (by alphabetical order)/ year of data collection/ Sample Size n: sex/gender (M/F) Age/grade (or mean age)	Country study design	Safety Questionnaire (perceived by) measured (actual)	Active behaviour tool/recall period/	The outcome measured Active Behaviour/	Neighbourhood	Examined variables Individual Family Neighbourhood	Spatial/ spatial statistical/ statistical analysis
1	[32]/- n = 473 (250 M/160 F) Aged 9–11 years old	United Kingdom Cross Section	Children questionnaire Self-reported (walking frequency + perception of the local environment + preferred travel method	From children questionnaire/ past 7 days	Walking frequency (high walkers/low walkers)	Local area	Sex/gender, race/ethnicity (White, Minority ethnic group, Asian, Black, Chinese, Mixed, Other, Not specified), Family characteristics (car ownership, number of rooms in the house)	Statistical analysis
2	[33], From 2001–2005 longitudinal study/ n = 170 (51% M) Aged 10–11 years	Australia Cross Section (CLAN) from a longitudinal study	Parents questionnaire Indices for (avoidance + defensive behaviour + perceived risk) and active transportation to 15 destination	Accelerometer/ 8 consecutive days non-school hours before and after school weekdays and weekend	*MVPA	Local area	Sex/gender. age	Statistical analysis
3	[34], T1 (April–July 2007)/ n = 1121(43%M) T2 (April-July 2008) n = 491 (39%M) Aged 9–10 years	United Kingdome Longitudinal Data from (SPEEDY) project	Parents perception survey (social/physical environment and rules regarding their children physical activity and perception of traffic safety concerns	Derived from children questionnaire on independent mobility	Independent mobility to school	Within 800 m pedestrian network buffer around the home (10 min walk)	Sex/gender Sociodemographic (cars ownership, parents’ education) + Environmental characteristics around the home and (within 100 m buffer of the shortest route to school	Spatial analysis to derive objectively environmental measures/ statistical analysis
4	[35]/- n = 492 (Sex/gender not reported)/ Aged 9–11-year-old	United Kingdom cross-section	From children (focussed group discussion)	From children (focussed group discussion)/-	Active Independent Mobility (AIM)	Local area	Sex/gender, age	–
5	[28]/ Sep.–Oct. 2009/ 35 children (18 M/17F)/ Aged 10–11 years	Finland cross-section	Parents questionnaire (children and parents mobility patterns + mobility licences, perceived safety)	GPS, mobility travel diary + individual interview/ 7 days	*active route to home	Buffer 500-m from home	Sex/gender IM licence Land use Types (using SLICES)	Spatial analysis
6	[36] /April2010 – May2011/ n = 736 (47% M and 52%F) included in the analysis/ Aged 10–12 years, grade 5–6	Canada Cross-section from [BEAT] project	Parents questionnaire (child outdoor active play + parents’ perception on the neighbourhood)	Accelerometer/ 7 days	*Outdoor playing time MVPA	Neighbourhood	Age, sex/gender, SES of the neighbourhood (neighbourhood income), neighbourhood perception (roads, personal safety, accessibility of facility)	Statistical analysis
7	[37]/During April and May of 2010 and 2011 n = 143 (49 M/94F) two groups aged 9–11 and 12–13 years/grade 5—8	Canada From the (STEAM) project	Parents questionnaire Children questionnaire (child habitual neighbourhood activities + mobility behaviour + environmental perception	GPS/ 7 days	Neighbourhood Activity Space (NAS)	AS 400, 800 m of home, the second set those found within 1,600 m Moore’s model	Sex/gender, age Environmental perception from child and parents + Neighbourhood type (land use) + Parents IM licences	Spatial analysis/ statistical analysis
8	[38]/ Between 2011 and 2012/ n = 254 (100 M/133F) aged 8–13 years (mean age of 10.5) and 239 parents	New Zealand Cross-section From (KITC) project	Parents questionnaire (CATI) (Demographics + neighbourhood perception + safety + social cohesion + connection + parental concerns), children IM	Travel Diary/ 7 days	Independent Mobility (IM)	The immediate street around the home	Sex/gender, age, + older sibling Parents demographics (sex ethnicity of (New Zealand European, Maori, Pacific Island, Samoan, Asian, Indian, Others), study or work outside the home, household (dwelling type, cars availability length of residency) + , IM + parents neighbourhood perception of safety + connect and cohesion,	Spatial analysis/ statistical analysis
9	[30] /-/ 735 parents of children (364 M/371F)/ aged 7–9 years in 9 schools returned the survey	Iran Cross-Section	Parents questionnaire (mode of transport in the previous week, demographics, access to school service and public transportation, attitude towards waking	Parents reported Perceived Walking Time to school (PWTS) in min	Perceived Walking to school	School to home area	Sex/gender, household characteristics (father/mother driving licence, owned cars, father/mother occupation status) + perceived safety of walking to school + school travel mode, parental attitude, walking time to school	Statistical analysis
10	[39]/2014/ n = 194 / aged 9–10 years -	United Kingdom cross-sectional	Parents Survey used NEWS_Y Index to derive perceived environment Children self-reported PA using PAQ-C	Self-reported PA derived from a questionnaire	Body Mass Index (BMI) /Self-reported PA	High and low deprived areas	Sex/gender, home environment (access to media in the bedroom, IM derived from parents’ questionnaires, Area level Deprivation*, perceived safety	Statistical analysis
11	[40]/ study between 2015–2016/ n = 458 (230 M/228F)/ aged 10–12	Canada Cross Section	Objective measures of Pedestrian safety Parents survey for perceived pedestrian safety	Accelerometer and GPS in the watch 7 days And activity log	Average of minutes per day of active outdoor play	1 km buffer zone around participants home	Sex/gender Race/ethnicity (white, non-white) Family characteristics: (single or dual parents’ household, number of siblings, household income, parental education, parents’ value of outdoor and income Pedestrian safety (traffic volume, traffic speed, traffic calming and pedestrian infrastructure	Spatial analysis/Statistical analysis
12	[41]/ Between 2011 and 2012/ n = 236 (104 M/132) for weekday analyses, and 210 (91 M/119F) for weekend days analyses. Age mean 9.8 for this study from 9 schools, grade 5–8	New Zealand Cross-section from (KITC) project	CATI-Parents questionnaire on neighbourhood perception using items from Ranui Action Survey + measured road network	Accelerometer + GPS + Travel diary/ 7 days outside school hours	*%MVPA	Buffer 1000-m around participants home address	Sex/gender, age, race/ethnicity, (New Zealand European, Maori, Pacific Island, Indian/Asian/Other Ethnicity) SES (car availability for pick up) + neighbourhood exposures (measured GIS street connectivity, distance to school, destination accessibility, Ratio of High-speed roads around school + streetscape audit)	Spatial Analysis/ Statistical Analysis
13	[42]/ - n = 830 parents of 4^th^ grade (412 M/418F)	United States Cross-section from [T-COPPE] longitudinal project	Parents questionnaire adapted from several surveys including the National Centre for Safe Routes to School Parents Survey, SPAN, (UH-PEAK), NEWS, and EnVivo) Personal safety + Traffic Safety	From parents’ questionnaire Inclusion criteria were that participants are within walking distance between home to school) GIS used to geocode participants address	Walking to school derived from National Safe Route To School Survey	Within walking distance of 3.2 km (using GIS and geocoded students’ home address	SES (car ownership, public assistance) race/ethnicity)	Spatial analysis to derive the area of exposures/ statistical analyses
14	[43]/ Between 2006 and 2008/ n = 1307 (639 M/661F)/ 10–11 years old from 23 schools	United Kingdom cross-section from (PEACH) longitudinal study	Children questionnaire (computerised) perception of the environment (aesthetic, nuisance, safety including traffic of places to cross, heavy traffic and road, social norm, constraints)	From the questionnaire Frequency in participation in active play, active travel and structured exercise and sport	Frequency of outdoor play, exercise and sport, active commuting Local-IM Area -IM	Not reported	Sex/gender, age, race/ethnicity (white, non-white, but not accounted in analysis) Perception of (Aesthetics, Safety, Social Norms, Nuisance, Constraints, accessibility, minutes of daylight from 3 pm till sunset), level of deprivation (using Index of Multiple Deprivation (IMD) and derived from seven categories of deprivation, Daylight, Pubertal status, BMI	Statistical analysis
15	[44]/Sep.-Dec.2014/ n = 144(72 M,72F)/ aged 7–12 years (mean age of 9.7 children)	United States cross section	Parents questionnaire (perception of the environment)	From the parents’ questionnaire	Active play	Walking distance 10–15 min	Sex/gender, age, race/ethnicity (Hispanic/Latino, African American, American Indian/Alaska Native, Asian American, White) Parents perception of built environment features Family SES	Statistical analysis
16	[45]/Between 2007–2009/ n = 145 (71 M/74F) / 6–11-years-old	United States Cohort cross-section study From (NIK) study	Parents questionnaire (demographics + prior victimization perception + stranger danger + crime perception) + Police reported crimes geocoded near participants home	Accelerometer/ 7 days	MVPA	Census blocks	Sex/gender, age, race, Household income, neighbourhood environmental walkability scale, collective efficacy, Prior crime victimisation survey, stranger danger and crime perception	Spatial analysis statistical analysis
17	[46]/ 2010/2011/ n = 354 (156 M) of grade 6^th,^ and their parents	Portugal cross-section from [SALTA] longitudinal study	Parents questionnaire (parental physical activity, family demographic, and perception (adapted from NEWS and previous studies) Children questionnaire to derive mobility style	Derived from a questionnaire of previous week physical activity based on IPAQ	Independent mobility (IM)	Local destinations	Sex/gender, age, family demography (parents age, education), parental PA, parents’ perception of neighbourhood safety (sidewalk, street safety, fear from strangers, crime and traffic safety)	Statistical analysis
18	[31]/the year 2009/ Grade 3 – 5/-	Iran Cross-section	Parents survey + Children survey on the perception of environmental factors that prevent children from walking to school	From the parents’ survey Differed the trips from home to school and from school to home	Walking to school	Home-school	Sociodemographic	Statistical analysis
19	[15]/- n = 190 (49%F) from two public schools/ aged 6–9(10) years old	Austria Cross-section	Parents questionnaire (demographic and household + parents mobility licences + mobility habits Child interview (understand IM motivation and licences)	From semi-structured questionnaire + Travel Diary (using KONTIV-format)	Active Independent Mobility (AIM)	Neighbourhood	Age, family background (working status of parents, vehicles per household, Parental attitude (promoters, pragmatists protectors) + IM licence	Statistical analysis
20	[29]/ Baseline collected in 2012 with three years follow up T1 n = 2108/50.5%F/ aged 5–11 years	United States Longitudinal study	Measured Crime Risk Index (CRI) from for each zip code from actual crime statistic	Height and weight assessed at baseline 2012 and three years later	Body Mass Index (BMI)	Urban Zipcode	Sex/gender, age, race/ethnicity (White, Asian, African American, Hispanic), demography (median household income and education) + Crime Risk Index, ^2^ Consumer Expenditure Data, the density of food outlet (using walk score and places for PA	Statistical analysis
21	[47]/-/ n = 291 (150 M/141F)/ aged 5–6 and n = 919 (424 M/495F) aged 10–12 from 19 primary schools	Australia cross-sectional	Parents & children questionnaire parents’ questionnaire (children’s walking and cycling and Perceived safety) compared to Children (perception of safety)	Walking and cycling trips from parents’ questionnaire	Frequent Walking and Cycling	Walking distance	Sex/gender Family background (language, SES, marital status, education, cars’ ownerships, own a dog Perception of parents (traffic, safety, pub. Trans) Perception of children (neighbourhood and view of parents)	Statistical analysis
22	[48]/ Fall of 2018/ n = 660 (315 M/341F) and their parents grade 5–8 of age 7 – 12 (mean age 9.5)	The Netherland Cross-section	Parents survey for safety perception	Derived from children survey (at school)	Travel mode to school	Participants were of Home-school Within 1 km distance	Sex/gender, age, Household (income, car ownership), weather, street connectivity	Statistical analysis
23	[49]/July – December 2007 n = 926 (463 M,463F) included in the analysis/ aged 10–12 years	Australia cross-section Data from (TREK) project	Parents questionnaire & children questionnaire Parents completed self-administered questions	steps count using Pedometer/7 days	Activity space IM index computed using children questionnaires	within 800 and 1600 m of child's home	Sex/gender, age, SES level of the school neighbourhood (Low, medium, high), maternal Education, IM index derived from parents and child questionnaires Parents and Child perception of safety, school-specific walkability (high/low), digitise pedestrian network	Spatial analysis/statistical analysis
24	[50]/between Jan. 2015 and Dec.2016/ n = 387(185 M/182F)/ aged 10–13 years (mean age 11.5)	Canada Longitudinal from Data from (Active Play Study)	Measured crime Report Against a person & Property For 24 months before measures	GPS/7 days	Average minutes per day of active transportation	Crime in 1 km road network buffer distance around participants home to define a neighbourhood	Sex/gender, age, race/ethnicity (White) parental education, family income and family profile, season, walkability index (using streets connectivity from the length of roads, intersection density, average block length, connected node ratio), proximity to destinations (walk score, distance to school, population density) and pedestrian safety from traffic	Spatial analysis/statistical analysis
25	[51]/- Children from 73 elementary school/-	United States Cross-section	Measured Crime Use geocoded Crime rate (8 major crimes index against the person + Traffic danger (crash rate)) to indicate Neighbourhood Safety Level	GIS derived Neighbourhood walkability Level from (estimate potential walkers, pedestrian facilities, residential density, land use mix, street connectivity)	Neighbourhood Walkability level (identify potential walkers)	School attendance areas	Race/ethnicity (Hispanic, Non-Hispanic, White) Poverty Derived—street walkability index, traffic danger, Neighbourhood-level walkability *Potential walkers (to school)	Spatial analysis/ spatial statistical analysis

*M* males, *F* Female, *(-)* data not reported, *BMI* Body Mass Index, *GPS* Global Positioning System, *NAS* Neighbourhood Activity Space, *CLAN* Children Living in Active Neighbourhoods, *SES* Socioeconomic status, *MVPA* Medium-to vigorous Physical Activity, *SPEEDY* Sport, Physical activity and Easting Behaviour Environmental Determinants in Young People, *BEAT* Built Environment and Active Transport, *GPS* Global Positioning System, *STEAM* Spatio-Temporal Exposure and Activity Monitoring, *KITC* kids in the City, *PAQ-C* Physical Activity Questionnaire for Older Children, *NIK* Neighbourhood Impact on Kids, *CRI* Crime Risk Index (measured crime using actual crime statistics), *TREK* Travel Environment and Kids Project, *CATI* Computer-aided Telephone Interview, *KIC* Kinds in the City, *T-COPPE survey* Texas Childhood Obesity Prevention Policy Education project, *IMD* Index of Multiple Deprivation is a composite score based on seven categories of deprivation (income, employment, health and disability, education skills and training, housing and geographical access to service), *PEACH* Personal and Environmental Association With Child’s Health, *SPAN* School Physical Activity and Nutrition, *UH-PEAK* Urban Hispanic Perceptions of Environment and Activity Among Kids, *En Vivo* TV reduction intervention study, *NEWS* Neighbourhood Environment Walkability Scale, *NEWS-Y* Neighbourhood Environment Walkability Scale for Youth used to assess parental perceptions of neighbourhood design, *SALTA* Environmental Support for Leisure and Active Transport, *KONTIV* format of travel diary survey for non-home activity patterns, *GIS* geographic information systems, *IPAQ questionnaire* International Physical Activity Questionnaire, *Local-IM* destinations of best friend’s house, school, local shops and park or playground, *Area-IM* destinations of swimming pool, library, cinema, arcade, bus stop, sports and shopping centre, *SWI* School Walkability Index derived from network connectivity and traffic volume, Neighbourhood-level walkability index derived from an (estimate of potential walkers, pedestrian facilities, residential density, street connectivity, land use mix), *Neighbourhood-level safety* derived from (traffic danger and the crime rate in a year), *TREK* Travel Environment and Kids

Studies denoted with * = Study measures and analysis accounted for day type (weekend/weekdays and outside school i.e. before and after school hours)

### Study methodological quality assessment

Differences between the first reviewer (RZ) and the second reviewer (BJ), in the output of the methodological quality assessment, was resolved via discussion to achieve full agreement. Of the twenty-five studies included, 8/25 studies were rated robust (32%), 14/25 studies (56%) were fair, and three studies (12%) were weak. Further details are in Additional file 2: Table 2.

### Measuring COAMB or health indices

**Fig. 2:**
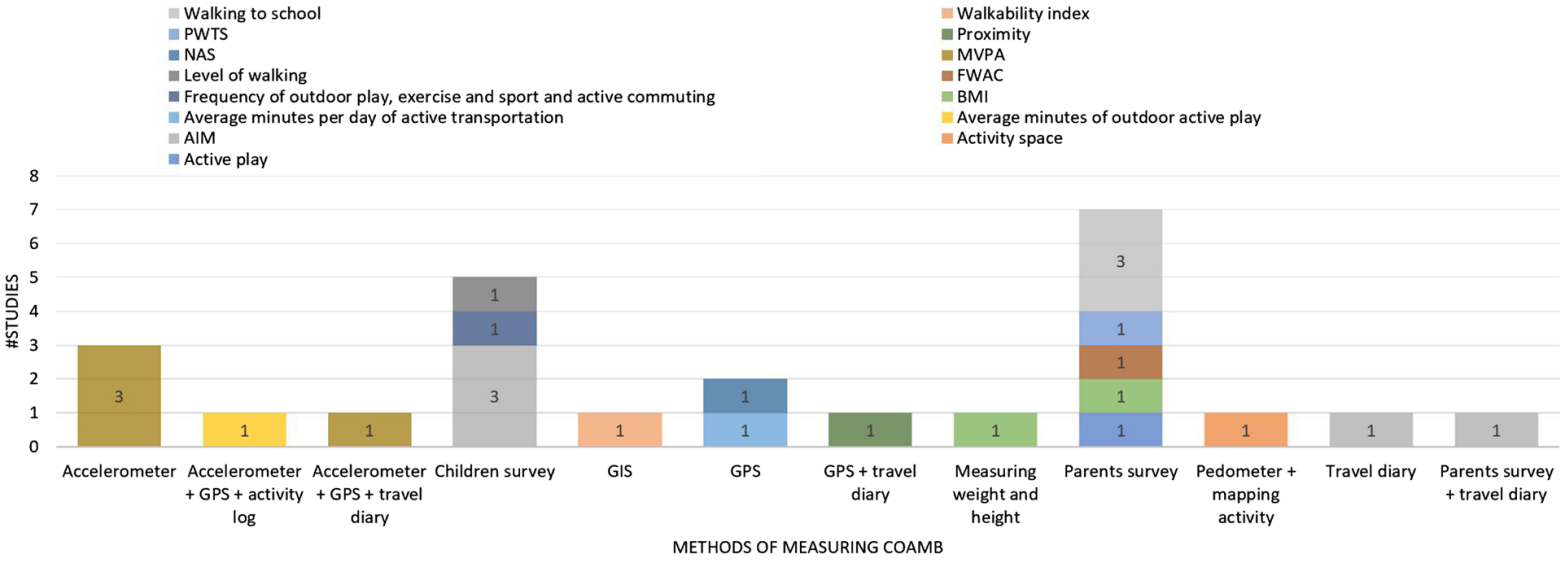
Means of measures and the outcome indices of children’s active Mobility behaviour. AIM = active independent mobility, BMI = body mass index, FWAC = frequent walking and cycling, MVPA = moderate-to-vigorous physical activity, NAS = neighbourhood activity space, PWTS = perceived walking to school, GPS = global positioning system, GIS = geographic information system

Over half of the studies (13/25 studies; 52%) obtained forms of COAMB from parent questionnaires and/or travel diaries. The remainder (12/25 studies; 48%) used objective measures, including 2/25 studies (8%) used body mass index (BMI) measures, with remaining used an accelerometer activity tracker in five studies (two Actigraphs, two Acticals, and two GT1M Actigraphs), an Accusplit pedometer (AH 120M8 KS10), a GPS spatial location tracker in five studies (two Garmin Forerunner 220 s, VGPS-900, GSM22, and QStarz BT-Q1000/BT-Q1000XT), and an ArcGIS to derive walkability index in one study. Some studies used a single tool such as an accelerometer [33, 36, 45] or GPS [50, 52] while a few studies combined more than one, such as a GPS with an accelerometer and travel diary/activity log [40, 41]. Variation of utilised tools and the discrete output units across studies are illsutrated below, Fig. 2.

#### Outcomes

The temporal characteristics of COAMB (weekends vs. weekdays and time of day, such as before and after school) were accounted for in 4/25 studies (16%) [28, 33, 36, 41]. Dissimilarities in the tools and methods used to measure forms of COAMB, as displayed in Fig. 2, followed by disparity in outcome being measured in different units across studies:

- Activity intensity: Moderate-to-vigorous physical activities (MVPA) was used in five studies. Step count from accelerometers was used in three studies [33, 36, 41, 45], while another study used parent questionnaires [44] to calculate children’s active play (both by frequency and duration of participation in vigorous-intensity or moderate-intensity activities and by reaching 60 min of daily PA based on the recommended guidelines). Another study depicted intensity by measuring children’s daily average minutes of outdoor active play [40].
- Activity space: Two studies used children’s movements across space to determine the size and geometry of movements when visiting places. The activity space is depicted by an area feature (closed polygon) and obtained by collecting multiple longitude and latitude points of active mobility. Circular multi-buffer rings represent various spaces, and each ring is built from the frequency with which children visited the places and the time spent there [52]. Alternatively, a minimum convex polygon connecting at least three points of the visited destinations is used to depict the activity space [49].
- Active route (derived path of the active travel between destinations): One study used GPS waypoints embedded in watches to derive a linear feature representation of active child transportation (i.e., the routes or path that a child uses to connect to his/her target destination) [28]. A longitudinal study by Vonderwalde also used GPS to identify active transportation trips made by children [50].
- Walkability indices: Objectively derived as the index of neighbourhood-level walkability estimates for potential walkers by combining land use mix, street network connectivity, residential density and pedestrian facilities [51].
- Health indices: Body mass index “the ratio of an individual's weight in kilograms divided by the height in meters squared (BMI = kg/m^2^)” to measure of body fat percentage and obesity [53]. In this review, one study measured BMI [29] and another used parents’ reported children’s weight and height [39].

In the remaining 13 studies, COAMB was assessed using a variety of subjective measurement methods:

- Walkability indices: Subjectively derived walking frequency from parent questionnaires [32].
- Home-school active travel: Studies used parent questionnaires and/or travel diaries to assess the perception of walking to school [30, 31], frequency of walking and cycling to school [47], frequency of walking to school [42] and satisfaction with active travel to school or mode of travel to school [48].
- Type of outdoor active behaviour: One study used the frequency of outdoor play, exercise, sport and active commuting [43]. Active independent mobility (AIM) was primarily derived by asking children for a list of destinations they are allowed to go to on their own or with a friend without an adult [15, 34, 35, 38, 46]. However, one study by Page et al. distinguished between two types of IM, namely Local IM and Area IM [43].

Additionally, a range of various technical data capturing specifications were depicted in the measures. For example, epochs, which is the interval of time for capturing rapid transitions of an activity, varied among and within the tools used. Using global positioning systems, studies used one-second [52], five-second [28] and 10-s epochs resampled to 30-s [45], and 15-s [40] epochs, while other manuscripts that used an accelerometer adopted 30-s epochs [41, 45]. Additionally, reviewed studies used a range of varied inclusion criteria (i.e. the minimum hours or days of measures to include for analysis; Table 2).

**Table 2:** Summary of variety in objective measures characteristics: organised by the output units, citation, a brief of the tools and inclusion and exclusions criteria

The output unit of measured COAMB	Citation	Tools used + duration in survey Data registry (epoch) Temporal measures (if present) (i.e. weekdays vs weekends or time segments of the day before and after school)	Inclusion/exclusion of collected GPS COAMB data
Intensity MVPA	[33]	Accelerometer for 8 consecutive days Epoch not reported during non-school hours Compared outside school hours for weekdays and weekends (before school [6 am] to school first bell) and evening (6–9 pm)	4 weekdays and at least 1 weekend for inclusion; data were excluded for any day if fewer than 10,000 and over 20 million steps counted to calculate MVPA
	[45]	Accelerometer for 7 days 30-s epochs –	10 h wear time for inclusion with no more than 20 min of consecutive zero counts and at least 3 days of accepted measures
	[36]	Accelerometer for 7 days 5-secs epochs Compared outside school hours for weekdays and weekends	10 h wearing time for 3 weekdays and 1 weekend day for inclusion in the analysis, MVPA > 3,581 counts per minute
	[41]	Accelerometer for 7 days Every 749/30-s epochs), + GPS 10-s epochs (cleaned and resampled to 30-s Compared outside school hours for weekdays and weekends (before school hours 8:00–8:45 am, after school 2:30–7:00 pm and weekends) + travel diary	Accelerometer outside school hours, 3 h or more of data (weekdays) and 7 h of data (weekend) GPS inclusion of data was not reported
Activity space	[49]	Pedometer for 9 days and mapping destination Epoch not reported –	Data with 1,000–30,000 steps daily included At least 4 days of data First and last day of measures removed from the analysis
Active transportation	[16]	GPS in a wearable watch for 7 days –	Data excluded with < 10 h GPS data and participants with < 4 days of 10 h GPS data (adopted from accelerometer standard measures)
Proximity	[28]	GPS + mobility diary for 7 days 5-s epochs –	No report of details
Neighbourhood activity space	[52]	GPS for 7 days Recorded each second Compared outside school hours for weekdays and weekends	3 h of out-of-school wearing time GPS (two weekdays) and 4 h of one weekend day Guided by accelerometer for inclusion
Active outdoor play (daily average)	[40]	GPS + accelerometer for 7 days 15-secsepochs	Remove < 10 h of accelerometer or GPS wear time or less than 15-s epochs

### Measuring the area of exposures to assess Impact on COAMB

Delineating the shared spaces that offer opportunities for the majority of daily routine activities is essential in children’s health research. The geography of environmental exposures must be captured to examine the association between variables within that specific area unit (called a neighbourhood) to explore the effect of exposures in the analysis. However, a concern that has been articulated in earlier research [54, 55] was reflected across reviewed studies in the diversity of measurement approaches and outcome units.

#### The outcomes

The measurement method adapted from earlier work [55], has been expanded to delineate how studies’ measurement techniques fall into one of five recognised types of measures. The output categories show the geographic context of the children assessed or measured by studies to derive safety exposures (Additional file 2: Figure 2). Under the most conventional approach within the scope of the studies (15/25 studies; 60%), the child’s neighbourhood was most often defined as the local area, the area within a 10-min walk, the street immediately near a child’s home, the walk between school and home, or the area of the school [15, 30, 32, 33, 35, 36, 38, 39, 42–44, 46, 47, 51]. Two studies (2/25; 8%) defined the neighbourhood as the administrative boundary of the census block or zip code, which falls under residence-based approach [29, 45]. Both the above categories are static and subjective representations. The activity buffer-based neighbourhood (3/25 studies; 12%) is a GIS-based unit of analysis obtained via buffers around a point feature (representing the home or school of each participant). However, this unit of analysis is depicted by different buffer radii (using visual inspection of the GPS waypoint and applying equal weight to the delineated buffer area) of 800–1000 m around the school [41]; or 400, 800, 1200 and 1600 [37], and 500 and 1000 m around the child’s home [28]. The activity space-based measure (1/25 studies; 4%) delineates the geometry of a child’s activity space and differs from the earlier group in that it is an irregular convex polygon composed of joining points (a minimum of three) representing the destinations visited by children [49]. A pre-defined buffer radius (using a distance based on earlier research findings such as 1 km around the participant’s home) can also depict the buffer-based neighbourhood (4/25 studies; 16%) [16, 34, 40, 42].

### Measuring safety exposures

Examined safety exposures in the neighbourhood reflected personal safety—either perceived (19/25 studies; 76%) or actual (3/25 studies; 12%)—or jointly addressed perceived and measured safety (3/25 studies; 12%). Road safety concerns were addressed in 15 out of 25 studies (60%), which in all but two studies were perceived rather than objective measures.

#### The outcome

Perceived personal safety was captured via questionnaires mailed to parents, administered through a computer-assisted telephone interview (parents or children) or conducted on school premises (children). A pre-designed set of questions that assess parents’ perceptions of environmental characteristics used: the Neighbourhood Walkability Index for Youth (NEWS-Y) instrument [39], the NEWS [46], Ranui Actions Survey [41], used a survey that was adapted from several earlier surveys [42], adapted questionnaires from a previous study [32], while the rest of reviewed papers have used questionnaires to fit the individuality of their research objectives. The respondents were asked about their own fear of being a victim of a crime in their neighbourhood [44], or to rate their agreement with statements about perceived potential crime/safety [45] and perceived neighbourhood risk [37]. Studies aimed at capturing general feelings of safety varied in their use of open-ended questions [44], close-ended 5-point Likert scales ranging from very unsafe to very safe [30], or 5-point Likert scales ranging from strongly agree to strongly disagree [41]. Of the 7/25 studies (28%) that assessed children’s perceptions, one examined how children perceive their parents’ view of safety and its effect on encouraging or discouraging active mobility [47].

Studies that assessed road safety perception (13/25 studies 52%) captured parents’ perceived road safety in 10/13 studies (78%) and children’s perceptions in 5/13 studies (38%). Studies used various indices as a proxy for road safety, that can be grouped under the perception of signals on a busy road [44], the availability of a sidewalk [40, 42, 44], driving style in terms of fast drivers [36] or careful drivers who pay attention to pedestrians and cyclists [42, 48], heavy traffic [32, 40, 46–48], safe places to cross the road [42, 44, 46, 49], road pollution [43] or traffic danger [35].

Measured personal safety was examined by using crime types (against persons and/or property) in two studies. However, these were separated in the analysis in one study [50] but combined in another [45]. Remaining studies used indices of; a crime risk index (computing the likelihood of a crime occurring in a neighbourhood based on actual crime statistics) [29], a neighbourhood safety level index (combining crime data with traffic volumes, the percentage of high-speed streets and accident rates) [51]. Objective measurements of road safety employed GIS to measure traffic speeds around schools, and they adopted road hierarchy as a proxy to derive the ratio of high-speed roads around schools [41] in combination with streetscape audit (via Google Street View). Another study generated indices of traffic volume, traffic speed and traffic calming as well as pedestrian infrastructure [40].

Controlled confounders of the same questionnaires in terms of individual characteristics were mainly child sex/gender (20/25 studies; 85%), race/ethnicity (9/25 studies; 36%) and family characteristics as a proxy for the socio-economic status (18/25 studies; 72%). However, ethnic/racial categories were inconsistently classified across studies, although some studies originated from the same country [29, 40, 44, 45, 51], Table 1. Indices used to address socio-economic status varied across studies, with car ownership followed by family income being the most discussed.

### Methodological considerations in current measures

**Fig. 3:**
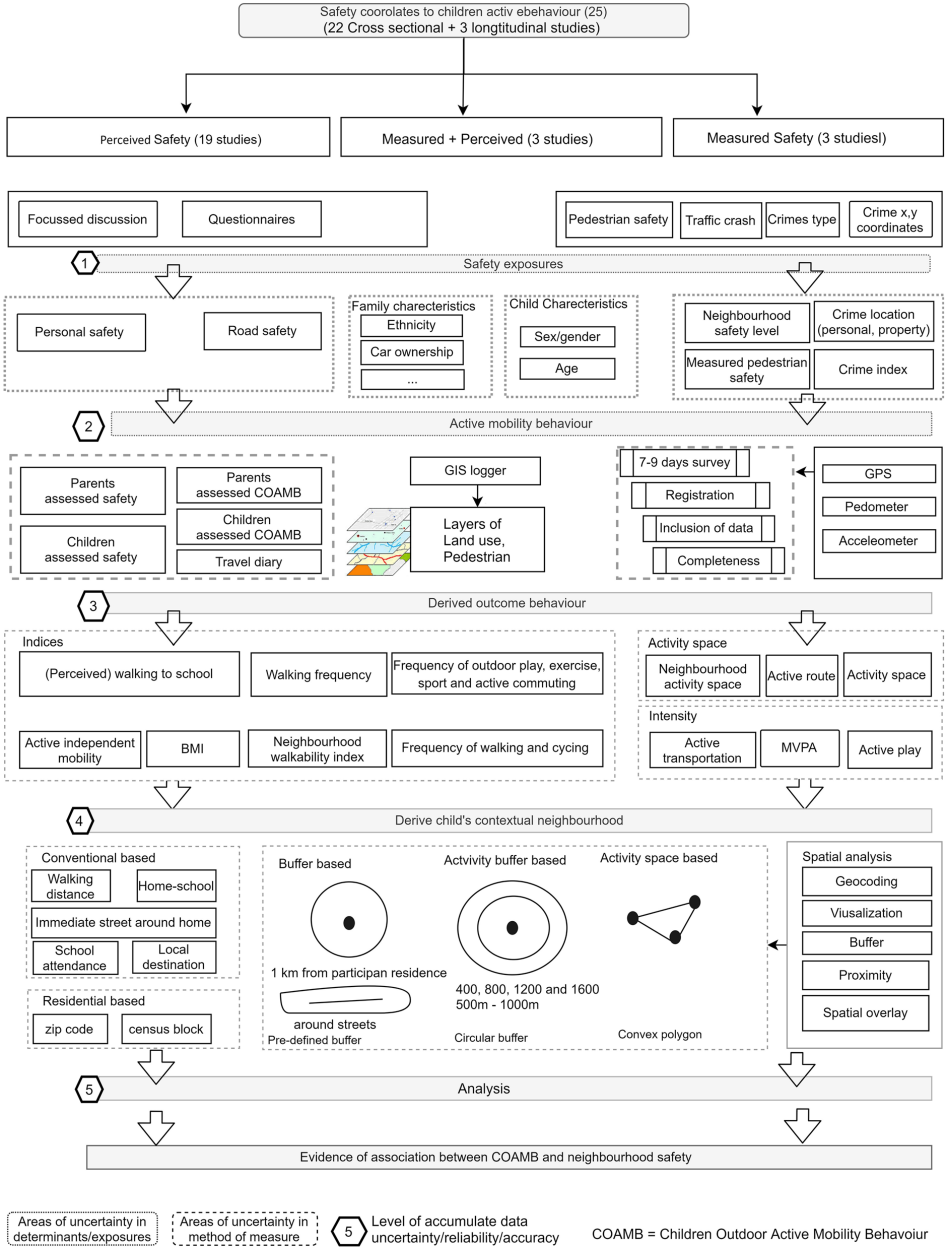
Schematic representation summarising the existing measurement methods in the studies included in the review

Overall, the review in findings revealed the existence of significant heterogeneity across studies in terms of what we are measuring (active behaviour, exposures and cofounders) and method of measure (data). Thus, to propose alternatives to guide future research, we delve into current methodological measurements issues. We illustrate schematically current methodological practices in layers based on the merit of two components: the determinants (in the dotted line); the methods of measure (in dashed line). Accumulated inconsistencies in what we measure and the uncertainty in how we measure increases the error of correlates and positional behaviour. Ignorance of the influences of reliability and the accuracy of data (levels 1–5, Fig. 3) poses a serious limitation as it may result in the misevaluation or misplacement of exposures and bias in evidence for policymakers. In general, in the majority of reviewed studies, a cross-sectional design was used, meaning the studies represented a short period of data collection (registry) that was not a real representation of the spatiotemporal behaviour of child mobility [43].

#### Consistency of measures

In level 1 of Fig. 3, we found an inconsistent accounting for sex/gender and age and race/ethnicity variation across studies. Additionally, we found that research capturing parents and or children’s views of safety are faced an inconsistent subgrouping of race/ethnicity or family socio-economic indicators preventing comparison across studies.

In the measure of safety exposures, evidence indicates variations between parents’ and children’s perceptions [7, 56, 57]. Despite that, the majority of studies relied on one side view of perceived safety (the parents). Thus, suggesting that an understanding of both parents’ and children’s perceptions of fear of safety may be incomplete. Additionally, perceived safety, as measured in self-reported questionnaires, are static mostly and lack more specific understating such as the intensity and frequency of feeling unsafe[13], or the geographic linkages to fear. In the actual personal safety measures, variations in means of using real crime data were depicted across studies. Earlier research agrees that different police-reported crime types produce different effects on various travel options [45, 58]. Additionally, except two studies [40, 45], an understanding of the variation between perceived and actual safety is lacking.

#### Reliability of measures

In level 2 of Fig. 3, included studies used questionnaires for either parents or their children to recall general feelings for safety perception as well as the child active behaviour. Daily travel diaries collected a child’s daily destinations visited and the mode of transportation. Both techniques rely on respondents to recall a particular event from a specific time, introducing human recall error or missing data that may impact analysis of the predictors of PA [19]. For example, a review by Kelly et al. 2013, on studies comparing self-reported and GPS-measured journey duration, concluded that participants were consistently over-reporting the duration of the journey. This suggests that when studies use self-reported journey behaviour, the journey duration should be treated as an overestimate [59] that agree with the findings of Klous et al. [60], 2017 for the rural population. The comparison between questionnaires to accelerometer by Määttä et al. [61], in specific to children, has as well suggested that accelerometer is a better measure than questionnaires when addressing PA duration among 11 years old children out of school PA.

Another significance observation found in most studies reviewed is that they lacked understanding of the temporality characteristics in the exposures as well as in the outcome of active behaviour. Such a characteristic of behaviour is confirmed in the review by Brooke et al. [62], on school-aged children PA. Thus, ignorance of the temporal component may lead to over- or under-estimated impact of exposures [55].

#### Accuracy of measures

Despite the proven advantages in the emerged tracking tools such as accelerometers and spatial technology of GPS offering the location and dimension of active mobility, studies often recognised some drawbacks that are depicted in level 3, Fig. 3:

##### Inclusion criteria

Majority of studies seem to operate within the timeframe of 7 to 9 consecutive days of a survey. However, varied inclusion criteria were adopted in each study, Table 2. Research using GPS was faced with the absence of a standard operating protocol for GPS device usage and thus adapted previous practices and established protocols for the accelerometer. The nature of the GPS data differs from accelerometers, and further guidelines on GPS protocol are warranted.

##### Registration

Variations in the epochs (frequency in seconds of capturing location) used in data registry across studies, as shown in Table 2, reflect the individuality of measures. In addition, the increased use of GPS to obtain high-resolution spatiotemporal data was faced with signals that are weakened or cut off when indoors [37], in urban settings, inside buildings, near trees and on cloudy days, thus resulting in errors as well as the battery life limitations. Although a new generation of GPS technology may facilitate the indoor GPS option, future research that intends to use such tools must give careful attention to study area structure to involve other supporting methods.

##### Completeness

Missing data in studies that used GPS or accelerometer devices were generally due to recruited study participants not wearing the device (forgetting to wear, not charged or turned off) during some parts of the day or had incomplete travel diary data [28]. Missing data means the 7 days measures include a significantly shorter period of data in the analysis and that visited destinations or trips are missed [63].

##### Confidentiality

This was not explicitly reported in reviewed papers, possibly due to the fact that the majority of studies that have used GPS to track children’s active mobility were being undertaken in developing countries. Nevertheless, existing evidence suggests that the perceived acceptability of such data collection method was lower in older low socio-economic population [65], as well as in specific ethnicity though more acceptance within the younger population [64] or in developing countries [66] where health technologies become less acceptable due to perceived privacy threats.

#### Uncertainty in representing the area of exposures (neighbourhood)

The current review portrayed the persistence of an important issue when using geographical data which is the articulated uncertain geographic context problem (UGCoP) [67]. As level 4 in Fig. 3 illustrates a variation in measurement methods and outcome across reviewed studies was found. Defining the geometry of the child neighbourhood to assess influences of environmental exposures ranged from subjective, arbitrary representations to an objective delineation. Buffers (circular) are a better representation of an individual’s mobility in space than subjective measures, yet they remain static in time. Additionally, circular proxies do not generally coincide with the area that children access. The minimum convex polygon, which measures activity space, is one of the prevailing spatial methods used to represent the geographical context in spatial epidemiology [54]. A recent study by Zhao et al. [54] argued the influence of the various methods of measurement in terms of multiple buffer radii or activity spaces (e.g., road network buffer, minimum convex polygon, weighted standard deviation eclipse) on analytical results related to health outcomes.

### A methodological conceptual framework to guide future research

Relevant data that we use in studies to assess the impact on children’s active health behaviour and outcomes are fundamental to research aimed at planning health intervention strategies. However, our review showed that researchers in this field currently lack agreement on not only what to measure but also primarily how to measure. In the past, several conceptual frameworks were developed to guide evidence-based research, yet the representation of influencing factors was either stationary and specific to one type of active behaviour (e.g. school active travel [7, 68]) or addressed a broad range of children’s ages (5–18 years old) [8]. The time element introduced by Pont et al. in the simplified framework [69] lacked spatial dimensions. Additionally, exposures that modify children’s active behaviour may very well be too complex to be depicted in one single framework [70]. Thus, a framework does not exist that expands beyond the identifications of variables to agree on the representation of exposures that modify primary school-aged children’s active mobility behaviour in this context. Based on the four identified levels of concern in Fig. 3, it is now crucial to conceptualise alternative measurement methods and technology to guide future studies.

**Fig. 4:**
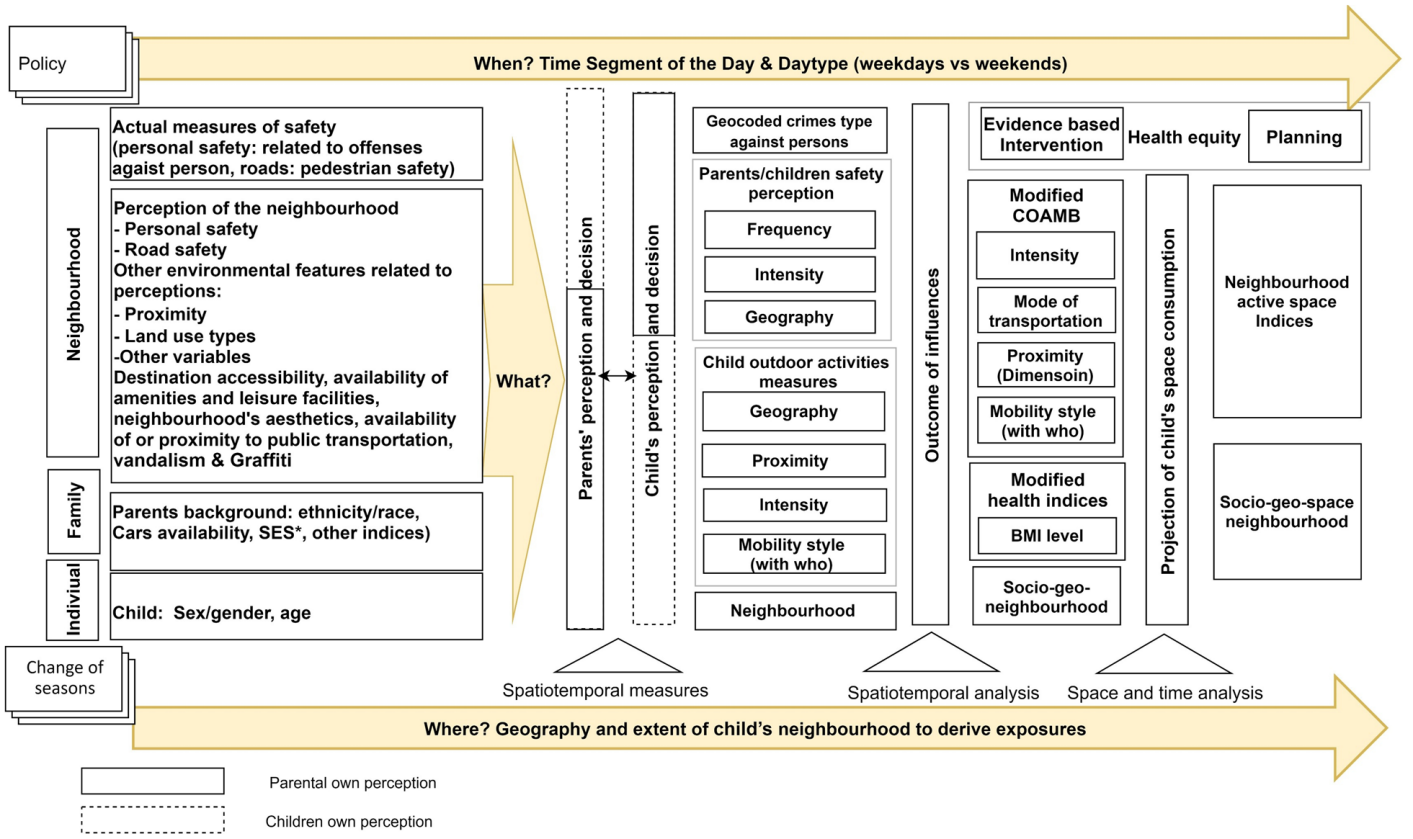
A methodological conceptual framework of children’s outdoor active mobility behaviour in a safety context. Exposures (on the left) are organised as per the socio-ecological model and by the output of measures (as we move right)

To guide future research, we proposed a triadic conceptual framework (see Fig. 4). In the framework, we distinguish three pillars (represented in yellow) that were derived from the theoretical representation of objects visualised by Peuquet, which is advantageous when examining human mobility in research [71]. We portrayed first the interplay of ‘what’ (determinants), which is arranged according to the socio-ecological domains of individual, family and neighbourhood influences in the context of safety. ‘Where’ addresses the spatial variable or dimension of behaviour influenced by safety. Finally, temporal characteristics (e.g. exposures and COAMB) answer the question of ‘when’ (i.e. the time of occurrences, such as weekdays vs. weekends, and the time segments of the day, frequency and regularity).

### Moving forward

Addressing public health concerns in the field of children and youth active mobility is expanding rapidly in terms of research conduct. However, despite technology advances, the challenge will likely, in the absence of standardisation, remain primarily in the data measures that may have an implication on the evidence. Results of the methodological quality appraisal showed that the majority of studies (88%) fall under robust or fair quality. Nevertheless, the heterogeneity in applied measurements and outcome extends across all studies. Thus, it is maybe crucial to draw upon the conceptual framework (Fig. 4) to achieve an outlook on alternative measurement methods that may reduce constraints portrayed in Fig. 3.

#### Study design

Future studies should aim to consistently account for gender, age and race/ethnicity differences in their measures as well as in analyses. A review of the evidence revealed that those variables have consistently predicted COAMB. In addition, a more comparable understanding of the effects of ethnicity and socio-economic status can be gained with standardisation of indices used and subgrouping. At the neighbourhood level, more research on understanding safety from children’s perspectives is warranted [56].

Additionally, understanding the population’s fears concerning types of crime may offer a more precise picture of the state of children*’* safety [72]. Thus, future studies would benefit from examining the impact of actual safety measures to consistently distinguish between types of crimes and to assess the most relevant crimes (e.g. crimes against persons and crimes against properties) to modify COAMB for appropriate interventions. Furthermore, despite findings in the literature confirming parents’ influences when perceiving low safety barriers, it is crucial to emphasise the dynamic nature of parents’ influence boundaries. Although gender-based, older primary school-aged children’s confidence in their neighbourhood (represented in dashed line, Fig. 4) results in children expanding the space they negotiate, and the impact of their parents’ perception (represented in solid line, Fig. 4) is seen to lessen. Finally, exploring the relationship between active behaviour and health indices, such as BMI, in the safety context is currently lacking. Thus, consistent accounting for BMI can help uncover this relationship in future research.

#### Methodology of measure

In the increased interest in evidence-based research about children’s active health to provide an appropriate baseline for targeting interventions, we suggest that future studies move away from subjective measures towards alternatives that offer a better output in research aiming for children health equity.

Regarding perceived safety measaures, we suggest that future studies replace the subjective and static understanding of safety perceptions to obtain more measurable feelings. The emergence of redesigned questionnaires about fear of crime in the last decade may help studies to (i) include specific geographical and temporal references for participants’ feelings [73, 74], (ii) capture the frequency and intensity of fear [13], and (iii) assign a timeframe for asking about feelings of worry (e.g. in the last year or the previous month) and perceived risk (e.g. in the next year, how likely…) [75].

Another important aspect is a consensus in future studies for representing the spatial and temporal elements of exposures (safety in this context), as well as the frequency and intensity of feelings. This is fundamental and may help researchers to answer a wider array of substantive research questions. Studies that intend to use questionnaires to capture safety perceptions may benefit from the location-based approach [76], which is likely to offer more comprehensive safety strategies. To overcome the human errors of recalling feeling safe, web-based applications have become more popular and can be installed on mobile devices for periodic access and instant recall of perceptions. For example, the use of a fear of crime mobile phone application that offers instant recording of people fear as welll as geolocating partcipants perception of concern [73, 74].

In measuring COAMB, the first triangle (Fig. 4) suggests that future studies consider the spatial (e.g. geographic location) and temporal (e.g. weekends and weekdays) elements in both reported and objective measures of COAMB. For studies that will be conducted in areas where there is perceived threat of confidentiality, potential GPS signal loss (dense urbanity) or limited budget, makes wearable technologies unattainable [66]. Thus, an alternative map-based questionnaire method has proven comparable to GPS in providing activity space information for exposure assessment [63]. To bypass errors caused by human recalling activities or daily diaries, future studies may consider taking advantage of the emerging web-based applications that can be installed on mobile devices or accessed periodically for instant recall of activities, such as the Finnish SoftGIS [77]. Other examples include the Public Participation GIS used for elderly active travel [78] or the Ecological Momentary Assessment mobile application used for health behaviour assessment [79].

In objective measures of COAMB at the foundation level, activity tracking and spatial technology offer a range of tools to collect, visualise and analyse the spatial and temporal outcomes of mobility behaviour. Moving forward, we suggest researchers take advantage of these tools and ensure that the temporality of active behaviour (in the survey and the analysis) over several days and at different times of the day is considered. However, standardisation in the protocols of measures using tools such as GPS and accelerometers may eliminate some of the studies’ mixed findings when using objective, rather than reported, measures of neighbourhood exposures and active behaviour.

Regarding the measure of children’s neighbourhoods, researchers are recommended to avoid assessing area of contextual exposures arbitrarily or measuring it in static space and time where possible. At the same time, to minimise the effect of the existing UGCoP (level 4, Fig. 3) in this conceptual framework, we suggest alternatives (see the second and third triangle in Fig. 4). The growing body of evidence confirmes the variation of safety impact on children’s activity space(s) according to gender and age [49], in the distance covered [28], and trips made [15, 33, 47, 52, 80]. Thus, we argue the need in future studies for innovative geo-approach and tool to enhance the accuracy when measuring environmental exposures’ effect. A socio-geo-neighbourhood, for example, that accounts for children’s gender and age and predict their likelihood of mobility within a space may be deemed fundamental. Older children require a different type of environment to be active than younger children; thus, the area and nature of active space is dynamic and fluctuates by gender, age and perceived safety barriers. In considering this direction, it is critical to move away from circular buffers as they do not coincide with the human mobility pattern. Integration of road network with children socio-geo background could derive a more reliable area when deriving exposures. Recent advances in deep learning [81] proven advantageous in detecting roads network using high-resolution satellite images.

In the era of big data, derived human activities are embedded in the social aspects of our daily lives. This has provided powerful insights that can help researchers to expand the trajectory of this field. For example, Pappalardo and Simini recently presented a framework for an algorithm to reproduce real human spatiotemporal patterns from mobile data [82]. Furthermore, expanding the analytical capabilities of space–time (space and time triangle, Fig. 4) when capturing and analysing data may uncover hidden spatiotemporal patterns of safety (actual or perceived) and children’s active behaviour in a space–time approach. For example, the space–time budget was used to understand the interaction of the social environment with people’s crime propensity [83]. Thus, integration of GPS, GIS with space–time may aid researchers to explore more challenging areas such as to predict the spatial heterogeneity in children’s populations in environmental context and may help policymakers to better plan the safety and mobility needs of active children.

### Strengths and limitations

This review focused on specific topics and omitted some closely related issues that are worthy of further investigation. For example, studies that account for policies, and seasonal/weather variation were not considered. Similarly, analysis of correlates, such as spatial or statistical methods used (level 5 in Fig. 3), was beyond the scope of this review, but correlates are likely to lead to different inferences due to differences in the underlying assumptions of analysis techniques. Assessment of methods to (a) clean the GPS data collected on COAMB (e.g. PALMS, Google Fusion Table software [40, 41, 50]) or (b) audit features of the neighbourhood, such as streetscape (e.g. NZSPACE [41]), were not included in the remit of this review. In addition, this review used comprehensive combinations of keywords to capture all studies, but some papers may have been omitted due to the inconsistent terminology used among studies; we tried to mitigate this risk by examining bibliographies, but the risk of omission remained. Additionally, reports and thesis studies were excluded. Despite these limitations, this study is, to the best of our knowledge, the first to comprehensively synthesise the methods of measures of primary school-aged children’s COAMB in a neighbourhood safety context. The paper is pioneer in constructing schematic representation that highlight layers in current methodlogical practices and to put forward a three-dimensional framwork for future measures. This review is also the first to assess the quality of the included studies methodologically, to synthesise crucial existing methodological gaps and to outline a framework to guide future research. Another strength included studies covered a wide range of countries origin.

## Conclusion

This review is the first to comprehensively and systematically synthesis measurement methodologies of safety exposures that impact COAMB. After reviewing 25 studies, we identified mixed methodological designs and an absence of standardisation in measures that may have led to the current diversity in studies’ outcomes. Such disparity in research outputs may be reducing the significance of synthesised evidence. The methodological quality assessment that this review undertaken showed that most studies were of moderate or weak quality regarding their measurement methods. We also schematically outlined accumulated layers of heterogeneity in the studies’ method of measures that may affect data reliability. We argued that our constructed three-dimensional conceptual framework is vital to guide future research aiming to assess neighbouirhood exposures impact, such as safety, on COAMB. Moreover, we suggested potential alternative methodological measures, tools and solutions for studies that aim to provide children with equal active health opportunities. Despite advances in spatial technology, ignoring the uncertainty produced by the heterogeneity in current measurement practices may result in misevaluation or misplacement of exposures. Until this problem is resolved, significant evidence may be buried by these measurement and analysis methods.

## Supplementary Information

**Additional file 1.** PRISMA checklist.

**Additional file 2.** Details of included and excluded criteria. Details of used search key terms. The methodological quality assessment criteria used to appraise each study, including table 1 showing a list of criteria that each study was assessed against. Table 2 shows details of each total study score, the percentage accumulated, and the assigned level of quality (Robust, fair and weak).

**Additional file 3. **Excel sheet with the total number of studies that were excluded and the reason for exclusion.

## Data Availability

All data generated for synthesising methods during the systematic review are included in the published article, and its supplementary information.

## Abbreviations

COAMB: Children’s outdoor active mobility behaviour
SAT: School active transport
GIS: Geographic information system
GPS: Global positioning system
MVPA: Medium-to-vigorous physical activity
AS: Activity space
NAS: Neighbourhood activity space
FWAC: Frequent walking and cycling
AIM: Active independent mobility
BMI: Body mass index
NEWS-Y: Neighbourhood walkability index for youth
PALMS: Personal Activity and location measurement system
PPGIS: Public participation GIS
EMA: The Ecological momentary assessment
CATI: Computer-aided telephone interview
