# Supplementary material for: Children’s outdoor active mobility behaviour and neighbourhood safety: a systematic review in measurement methods and future research directions
Source: Int J Health Geogr. 2021 Jan 7;20:2. doi: 10.1186/s12942-020-00254-w (PMC7792091; doi:10.1186/s12942-020-00254-w)
Supplement: Supplementary file 2 — Additional file 2. Details of included and excluded criteria. Details of used search key terms. The methodological quality assessment criteria used to appraise each study, including table 1 showing a list of criteria that each study was assessed against. Table 2 shows details of each total study score, the percentage accumulated, and the assigned level of quality (Robust, fair and weak). [file 12942_2020_254_MOESM2_ESM.docx]

**Additional File 2**

**Detailed Inclusion and Exclusion Criteria**

Papers included in the study for review must be

- Peered review papers,
- Participants in studies were the population of primary school-aged children and/or their parents
- Examined impact of neighbourhood’s perceived safety (personal and roads danger) by parents and/or their primary school-aged children population on children active behaviour
- Reported or measured forms of children active mobility behaviour including walking, cycling, scooting, running) in terms of active travel to a destination, active play and exercise.
- Reported or Measured the temporal active mobility behaviour outside school hours (i.e. the time of before and after school or weekdays and weekends).
- Studies included if they reported a study objective, participant, methods of measures and analysis and output

Excluded papers if

- published in other languages than English
- mean age or age group of children addressed in a study is younger than 5 and older than 12 years old.
- the study addressed safety from the intervention view
- the study didn’t display clearly the research objective, study design, age group and result.

There were no limitations regarding the year of publications or geographic location or study design or sample size

**Detail of search terms:**

(1) target population “Children”, “youth” or “kids”, “young”, “primary school children” (2) active behaviour “active play”, “active mobility”, “physical activity”, moderate to vigorous physical activity”. “Outside School Active Behaviour, “active travel” (3) exposures of the social neighbourhood “perceived safety”, “social neighbourhood”, “neighbourhood safety”, “measured safety”, “perception of personal safety”, “road measures” (4) moderators “parents perception of safety” parents and children perception of safety” or “children perception”,” (5) spatiotemporal aspects: “spatiotemporal measures, geographic information systems”, “GIS”, time geography”, “space and time geography”, “objective measures” or “GPS”.

**Search strategy in ProQuest**

The document type is articles, limited to peer-reviewed publication, a date is all dates. The source type is articles. Language is in English. Keywords ( ( Children OR kids ) AND (active behaviour OR "physical activity " OR "spatial measures" OR "GPS " OR "active mobility” OR "spatiotemporal measures " ) AND ( Safety "crime" ) OR ( “road safety" ) AND (“neighbourhood”).

**Methodological assessments of individual studies**

# The comprised checklist in Table 1 to assess individual studies consist of six sections. The first three: Part A, B and C consist of four sub-items, each (0.25 point for each) that relevant to the quality of the study reporting. Each criterion was rated as yes = 0.25 point, whilst no points were assigned if the criterion was absent or inadequately described. The second part was specific to the methods of measuring active mobility, the area delineation of neighbourhood and safety. For active behaviour, the maximum score was 1 point.

Table 1 Criteria for methodological quality assessment and score for each item with a total score per subsection

| **Study** | **Criteria** | **Description** | **Quality score total** |
| --- | --- | --- | --- |
| **Characteristics of reporting quality assessment** | Part A | 1. **Study Objectives**: Were the study objectives clear? 2. **Design**: Was the study design appropriate for the research undertaken? 3. **Target Population:** Was the target population defined? 4. **Random Sampling:** Was a random sample of the target population taken or was sampling appropriate for the study design? | 0.25 point for each point if yes  Total maximum score = 1 |
|  | Part B | 1. **Study Participant Number** Was the number of participants at each stage reported? 2. **Participants Inclusion/exclusion:** Were criteria for inclusion and exclusion of participants in the analysis used? 3. **Study Population:** Was study population sufficiently described, i.e. sample size gender, age, indicators of socioeconomic status? 4. **Participant Recruitment:** Was participant recruitment described or referred to? | 0.25 for each point  Total maximum score = 1 |
|  | Part C | 1. **Response Rate:** Was the response rate of 60% or more? 2. **Data Collection**: Did the author describe data collection, i.e. by mail, by interview, objective measure? 3. **Data Sources:** Did the author describe the source of data, i.e. questionnaire, survey, focus group, accelerometer, GPS? 4. **Missing Data:**  Were number/percentage of participants with missing active behaviour data reported and did at least 80% of enrolled participants provide complete data to include in the analysis? | 0.25 for each point if yes  Total maximum score = 1 |
| **Methodological measures and analysis assessment** | Measures | 1. **Objective Measures:** Did the study objectively measure active mobility, i.e. activity tracking. Spatial technology or web application? 2. **Subjective Measure:** Were measures reliable and valid? | GPS = 0.25  (accelerometer) = 0.25  questionnaire = 0.25  Travel diary = 0.25  N/A  Total = between 0.25 -1 |
|  |  | 1. **Temporal Measures:** was the measured active behaviour related to the **temporal** characteristics, i.e. weekends vs weekdays or before and after school? | Yes = 0.5  No = 0  N/A  Total = 0.5 |
|  |  | 1. **Measures Perceived Safety Characteristics:** Did the study account for the spatial or the temporal features of safety feelings? (weekdays vs weekends or daytime (before and after school) Or was measured safety measured using geocoding? | Yes = 0.5  No = 0 |
|  |  | 1. **Area of Exposures:** Did the study delineate the exposure area "neighbourhood" objectively or assess arbitrarily? | Objectively = 0.5  Arbitrary = 0  N/A |
|  | Analysis | 1. **Analysis: W**as it clear what was used to determine statistical significance, e.g. p-value, confidence interval, OR was a spatial statistical method used? | Yes = 0.5  No = 0 |
|  |  | 1. **Output Evidence:** Did the analysis account for the spatiotemporal behaviour of the evidence? | Yes = 1  No = 0 |
|  | variables | 1. **Cofounders:** Did the study account for the cofounders of age, sex, ethnicity and family characteristics? | 0.25 for each point if yes  Total maximum score = 1 |

# The higher score is awarded when a combination of methods was employed (e.g. GPS and accelerometer and questionnaire or travel diary), the study will be awarded 0.75 points. The remaining characteristics of the temporality of active behaviour and safety, neighbourhood definition, and accounting for temporality in the analysis each collect (0.5 points). However, when a measure did not apply to the pre-defined checklist, it is discounted from the overall study sum of points of quality score. For example, a study used body mass index (BMI) as an outcome index for active health but didn’t involve temporal nature. Accounting for cofounders of age, sex/gender, race/ethnicity and socioeconomic status in the analysis each earn 0.25 point. The quality score was converted to a percentage by dividing each study quality score by the highest possible study quality score (adjusted by criterion measured) and multiplied by 100 to obtain study quality percentage (Schoeppe, Duncan, Badland, Oliver, & Curtis, 2013).

A study was rated as robust methodological quality with a percentage of more than ≥66.7%, as fair quality with a rating of ≥50-<66.6% and as a weak quality with a rating of <50%

Table 2 Output of the methodological quality assessment per a study in percentage and rated quality level (Robust, Fair, Weak)

| **Study citation** | **Study Objectives, design, target population, random sampling (0.25 each)** | **study participants, inclusion/exclusion, study population, participants recruitments (0.25 each, total 1)** | **Response rate, data collection, data sources, missing data. (0.25 each, total 1)** | **Did the active behaviour data collection was objectively measured? (0.25 for each method, total 1 point,)** | **Did the measure of active behaviour account for temporal characteristics? (0.5 point)** | **Has the perceived safety measured the temporal characteristics? Or has measured safety used geocoded data in actual crime for personal safety or in road safety? (0.5 point)** | **Did the study delineate the exposure area "neighbourhood" objectively (0.5 point)** | **Did the study evidence Accounted For spatiotemporal behaviour in output (1 point)** | **Was it clearly described the statistical method and to assess significance association or did the study describe the method of spatial analysis? (0.5)** | **Did the study account for the four cofounders (age, sex, ethnicity and family characteristics) (0.25 each, total 1 point)** | **Quality score total** | **%** | **overall rating of evidence Quality** |
| --- | --- | --- | --- | --- | --- | --- | --- | --- | --- | --- | --- | --- | --- |
| (Alton, Adab, Roberts, & Barrett, 2007) | 1 | 0.75 | 0.5 | 0.25 | 0 | 0 | 0 | 0 | 0.5 | 1 | 4 | 50.0 | **Fair** |
| (Carver, Timperio, Hesketh, & Crawford, 2010) | 1 | 1 | 0.75 | 0.5 | 0.5 | 0.5 | 0 | 1 | 0.5 | 0.5 | 6.25 | 78.1 | **Robust** |
| (Carver, Panter, Jones, & van Sluijs, 2014) | 1 | 1 | 0.75 | 0.25 | 0 | 0 | 0.5 | 0 | 0.5 | 0.75 | 4.75 | 59.4 | **Fair** |
| (Davis & Jones, 1996) | 1 | 0.5 | 0.25 | 0.25 | 0 | 0 | 0 | 0 | N/A | 0.5 | 2.5 | 35.7 | **Weak** |
| (Fagerholm & Broberg, 2011) | 1 | 0.75 | 1 | 1 | 0.5 | 0 | 0.5 | 0.5 | 0.5 | 0.5 | 6.25 | 78.1 | **Robust** |
| (Faulkner, Mitra, Buliung, Fusco, & Stone, 2015) | 1 | 1 | 1 | 0.5 | 0.5 | 0 | 0 | 0.5 | 0.5 | 0.75 | 5.75 | 71.9 | **Robust** |
| (Lin et al., 2017) | 1 | 0.5 | 0.75 | 0.25 | 0 | 0 | 0 | 0 | 0.5 | 1 | 4 | 50.0 | **Fair** |
| (Loebach & Gilliland, 2016) | 1 | 1 | 1 | 0.5 | 0.5 | 0 | 0.5 | 0 | 0.5 | 0.75 | 5.75 | 71.9 | **Robust** |
| (Mehdizadeh, Mamdoohi, & Nordfjaern, 2017) | 1 | 1 | 1 | 0.25 | 0 | 0 | 0 | 0 | 0.5 | 0.75 | 4.5 | 56.3 | **Fair** |
| (Timperio, Crawford, Telford, & Salmon, 2004) | 1 | 1 | 0.75 | 0.25 | 0 | 0 | 0 | 0 | 0.5 | 0.75 | 4.25 | 53.1 | **Fair** |
| (Noonan, Boddy, Knowles, & Fairclough, 2016) | 1 | 1 | 0.75 | 0.25 | 0 | 0 | 0 | 0 | 0.5 | 0.25 | 3.75 | 46.9 | **Weak** |
| (Nguyen, Borghese, & Janssen, 2018) | 1 | 1 | 0.75 | 1 | 0 | 0.5 | 0.5 | 0 | 0.5 | 1 | 6.25 | 78.1 | **Robust** |
| (Oliver et al., 2015) | 1 | 1 | 1 | 1 | 0.5 | 0.5 | 0.5 | 1 | 0.5 | 1 | 8 | 100.0 | **Robust** |
| (Oluyomi et al., 2014) | 1 | 1 | 1 | 0.25 | 0 | 0 | 0.25 | 0 | 0.5 | 0.5 | 4.5 | 56.3 | **Fair** |
| (Page, Cooper, Griew, & Jago, 2010) | 1 | 1 | 0.75 | 0.25 | 0 | 0 | 0 | 0 | 0.5 | 0.75 | 4.25 | 53.1 | **Fair** |
| (Roberts, Knight, Ray, & Saelens, 2016) | 1 | 1 | 0.75 | 0.25 | 0 | 0 | 0.25 | 0 | 0.5 | 0.75 | 4.5 | 56.3 | **Fair** |
| (Stephanie H. Kneeshaw-Price et al., 2015) | 1 | 1 | 0.5 | 0.5 | 0 | 0.5 | 0 | 0 | 0.5 | 0.25 | 4.25 | 53.1 | **Fair** |
| (Santos, Pizarro, Mota, & Marques, 2013) | 1 | 1 | 0.75 | 0.25 | 0 | 0 | 0 | 0 | 0.5 | 0.5 | 4 | 50.0 | **Fair** |
| (Shokoohi, Hanif, & Dali, 2012) | 1 | 0.5 | 0.5 | 0.25 | 0 | 0 | 0.25 | 0 | 0.25 | 0 | 2.75 | 34.4 | **Weak** |
| (Stark, Frühwirth, & Aschauer, 2018) | 1 | 1 | 0.75 | 0.25 | 0 | 0 | 0 | 0 | 0.5 | 0.5 | 4 | 50.0 | **Fair** |
| (Suminski, Robson, May, Blair, & Orsega-Smith, 2018) | 1 | 1 | 1 | N/A | N/A | 0.5 | 0.5 | 1 | 0.5 | 1 | 6.5 | 108.3 | **Robust** |
| (van den Berg, Waygood, van de Craats, & Kemperman, 2020) | 1 | 0.75 | 1 | 0.25 | 0 | 0 | 0.25 | 0 | 0.5 | 0.5 | 4.25 | 53.1 | **Fair** |
| (Villanueva et al., 2012) | 1 | 1 | 0.75 | 1 | 0 | 0 | 0.5 | 0 | 0.5 | 0.75 | 5.5 | 68.8 | **Fair** |
| (Vonderwalde, Cox, Williams, Borghese, & Ian Janssena, 2019) | 1 | 1 | 1 | 1 | 0 | 0.5 | 0.5 | 0.5 | 0.5 | 1 | 7 | 87.5 | **Robust** |
| (Zhu & Lee, 2008) | 1 | N/A | N/A | 0 | 0 | 0.5 | 0.5 | 0 | 0.5 | 0.5 | 3 | 50.0 | **Fair** |

**Figure 1. Distribution of Studies by country of origin and the type of safety addressed.**

**Figure 2. Studies grouped by neighbourhood measurement approaches**
